# Validation of the French version of the alcohol, smoking and substance involvement screening test (ASSIST) in the elderly

**DOI:** 10.1186/1747-597X-7-14

**Published:** 2012-06-20

**Authors:** Riaz Khan, Anne Chatton, Gabriel Thorens, Sophia Achab, Audrey Nallet, Barbara Broers, Gerard Calzada, Vladimir Poznyak, Daniele Zullino, Yasser Khazaal

**Affiliations:** 1 Division for Addictology Department of mental health and psychiatry University Hospitals, Geneva University, Geneva, Switzerland; 2 Department of Primary care and community medicine, University Hospitals, Geneva University, Geneva, Switzerland; 3 Department of Mental Health and Substance Abuse, World Health Organization, Geneva, Switzerland

**Keywords:** Addiction, Substance use disorders, Tobacco, Alcohol, ASSIST, Elderly

## Abstract

**Background:**

Substance use disorders seem to be an under considered health problem amongst the elderly. The Alcohol, Smoking and Substance Involvement Screening Test (ASSIST), was developed by the World Health Organization to detect substance use disorders. The present study evaluates the psychometric properties of the French version of ASSIST in a sample of elderly people attending geriatric outpatient facilities (primary care or psychiatric facilities).

**Methods:**

One hundred persons older than 65 years were recruited from clients attending a geriatric policlinic day care centre and from geriatric psychiatric facilities. Measures included ASSIST, Addiction Severity Index (ASI), Mini-International Neuropsychiatric Interview (MINI-Plus), Alcohol Use Disorders Identification Test (AUDIT), Revised Fagerstrom Tolerance Questionnaire-Smoking (RTQ) and MiniMental State(MMS).

**Results:**

Concurrent validity was established with significant correlations between ASSIST scores, scores from ASI, AUDIT, RTQ, and significantly higher ASSIST scores for patients with a MINI-Plus diagnosis of abuse or dependence. The ASSIST questionnaire was found to have high internal consistency for the total substance involvement along with specific substance involvement as assessed by Cronbach’s α, ranging from 0.66, to 0.89 .

**Conclusions:**

The findings demonstrate that ASSIST is a valid screening test for identifying substance use disorders in elderly.

## Introduction

Despite the importance of substance use disorders amongst the elderly and possible late onset of these disorders, it seems that this health issue is underscreened and frequently unnoticed in clinical settings [1-6]. This appears to be of concern for substance use impact on general and mental health. Furthermore, the pattern of drug use has changed, during the last decade, with an increasing illicit drug involvement in older adults seeking substance abuse treatment [2].

The World Health Organization (WHO) has identified tobacco, alcohol, and prohibited drugs use as amongst the top20 risk factors of ill-health [7]. This consideration has led the WHO to adopt a public-health approach for the issue, aiming to improve screening for substance use as well as early intervention for the problem [7].

To achieve the above mentioned aim, it is crucial to develop a reliable and user friendly screening instrument. In fact, the existing screening instruments have several limitations for the use in primary care settings [8]. For example, the Addiction Severity Index (ASI) [9], is time-consuming to administer in primary care settings. Furthermore, briefer instruments, such as the CAGE-Adapted to Include Drugs [10], have a focus on dependence, which is less useful for detecting problematic or risky substance use in nondependent persons.

Moreover, self report screening tests have limitations from a cross-cultural perspective. In fact, most were developed in the United States of America and do not have documented sensitivity and specificity for use in other cultures, and have not been extensively validated as well.

In order to overcome these difficulties, WHO developed, The Alcohol Use Disorders Identification Test (AUDIT), a brief, widely used and validated instrument in primary and mental health facilities [11,12].

In order to enlarge this successful approach to other substances, WHO developed The Alcohol, Smoking and Substance Involvement Screening Test (ASSIST) . The instrument aims to screen for problematic or risky substance use (available versions: http://www.who.int/substance_abuse/activities/assist/en/index.html).

ASSIST (ASSIST V3.0) consists of eight questions covering tobacco, alcohol, cannabis, cocaine, amphetamine type stimulants, inhalants, sedatives, hallucinogens, opiates and ‘other drugs’. Question 1 assesses lifetime use of substances, and the second question deals with the frequency of substance use during the last 3 months. Answers to this question are rated on a 5-point frequency scale ranging from ‘never’ (in the past 3 months) to ‘daily or almost daily’. If none of the substances have been used in the past 3 months, the interviewer can skip to the last three questions about problems and former usage patterns in their lifetime. If any substance has been used during the past 3 months, questions 3–5 are asked, before concluding with questions 6–8. Question 3 explores the compulsion to use substances in the previous 3 months. This is a measure of psychological dependence. Question 4 screens the domains of health, social, financial or legal problems associated with substance use within the past 3 months. Question 5 explores whether participants have failed to meet role obligations. Questions 6–8 screen lifetime and recent problems, including relatives; concerns about previous attempts to control substance use and current or lifetime injection of drugs.

Several studies carried out around the World found that ASSIST items were reliable and that the ASSIST screening procedure was feasible in primary care settings [13] as well as in psychiatric settings [14]. It was repeatedly found that ASSIST scores have significant correlations with several other measures, such as the Addiction Severity Index-Lite (a short version of ASI), Severity of Dependence Scale and AUDIT. Furthermore, the instrument has a good discriminative validity established on its capacity to discriminate between substance use, abuse and dependence for the substances tested. The scores derived from ASSISTV3.0 are presented in Table 1 and as presented elsewhere [14]. The specific formulae for each domain together with the maximum score for this domain are shown in the last column of the table.

**Table 1:** ASSIST V3.0: domain description, formula of each domain and domain maximum score

**Domain Label**	**Description of Domain/Score**	**ASSIST Formula**
**1A**	Lifetime substance use – including alcohol & tobacco	∑ Q1a + 1b + 1c + 1d + 1e + 1f + 1g + 1h + 1i + 1j (Max Score: 30)
**1B**	Lifetime illicit drug use – excluding alcohol & tobacco	∑ Q1c + 1d + 1e + 1f + 1g + 1h + 1i + 1j (Max Score: 24)
**2A**	Global continuum of substance risk – including alcohol & tobacco	∑ Q1a – j + 2a – j + 3a – j + 4a – j + 5a – j + 6a – j + 7a – j + 8 (Max Score: 422)
**2B**	Global continuum of illicit drug risk – excluding alcohol & tobacco	∑ Q1c – j + 2c – j + 3c – j + 4c – j + 5c – j + 6c – j + 7c – j + 8 (Max Score: 338)
**3A**	Specific Substance Involvement – Tobacco (or ASSIST tobacco score)	∑ 2a + 3a + 4a + 6a + 7a (Max Score: 39)
**3B**	Specific Substance Involvement – Alcohol (or ASSIST alcohol score)	∑ 2b + 3b + 4b + 5b + 6b + 7b (Max Score: 39)
**3C**	Specific Substance Involvement – Cannabis (or ASSIST cannabis score)	∑ 2c + 3c + 4c + 5c + 6c + 7c (Max Score: 39)
**3D**	Specific Substance Involvement – Cocaine (or ASSIST cocaine score)	∑ 2d + 3d + 4d + 5d + 6d + 7d (Max Score: 39)
**3E**	Specific Substance Involvement -ATS (or ASSIST ATS score)	∑ 2e + 3e + 4e + 5e + 6e + 7e (Max Score: 39)
**3F**	Specific Substance Involvement – Inhalants (or ASSIST inhalants score)	∑ 2f + 3f + 4f + 5f + 6f + 7f (Max Score: 39)
**3G**	Specific Substance Involvement – Sedatives (or ASSIST sedatives score)	∑ 2g + 3g + 4g + 5g + 6g + 7g (Max Score: 39)
**3H**	Specific Substance Involvement – Hallucinogens (or ASSIST hallucinogen score)	∑ 2h + 3h + 4h + 5h + 6h + 7h (Max Score: 39)
**3I**	Specific Substance Involvement – Opioids (or ASSIST opioid score)	∑ 2i + 3i + 4i + 5i + 6i + 7i (Max Score: 39)
**3J**	Specific Substance Involvement – Other	∑ 2j + 3j + 4j + 5j + 6j + 7j (Max Score: 39)
**4A**	Total Current Frequency of Substance Use – including alcohol, *excluding tobacco & ‘other drugs’	∑ Q2b – i (Max Score: 48)
**4B**	Total Current Frequency of Illicit Drug Use – *excluding alcohol, tobacco & ‘other drugs’	∑ Q2c – i (Max Score: 42)
**4C**	Current Frequency alcohol use	Q2b (Max Score: 6)
**4D**	Current Frequency cannabis use	Q2c (Max Score: 6)
**4E**	Current Frequency cocaine use	Q2d (Max Score: 6)
**4F**	Current Frequency amphetamine use	Q2e (Max Score: 6)
**4G**	Current Frequency inhalant use	Q2f (Max Score: 6)
**4H**	Current Frequency sedative use	Q2g (Max Score: 6)
**4I**	Current Frequency hallucinogen use	Q2h (Max Score: 6)
**4J**	Current Frequency opioid use	Q2i (Max Score: 6)
**5A**	Dependence – all substances including alcohol & tobacco	∑ Q1a – j + 2a – j + 3a – j + 6a – j + 7a – j (Max Score: 270)
**5B**	Dependence – illicit drugs excluding alcohol & tobacco	∑ Q1c – j + 2c – j + 3c – j + 6c – j + 7c – j (Max Score: 216)
**6A**	Abuse – all substances including alcohol & tobacco	∑ Q1a – j + 2a – j + 4a – j + 5a – j + 6a – j (Max Score: 300)
**6B**	Abuse – illicit drugs, excluding alcohol & tobacco	∑ Q1c – j + 2c – j + 4c – j + 5c – j + 6c – j (Max Score: 240)

The French version of the ASSIST was recently validated in a psychiatric and primary care setting [14]. ASSIST V3.0 to our knowledge has not been validated amongst the elderly subgroup. The present study aims to assess the psychometric properties of the French version of ASSIST in an elderly sample.

## Methods

### Participants and procedure

One hundred participants were recruited in the University Hospitals of Geneva between January 2010 and June 2010. The study was systematically proposed to the patients who attended the treatment facility during the study period until reaching the inclusion of 100 participants. Forty-two subjects were recruited from clients attending the geriatric policlinic day care centre of the Department of Community Medicine (a geriatric primary care outpatient facility). Fifty-eight patients were recruited from geriatric psychiatric day-hospital.. Participants from the geriatric primary care outpatient facility were mostly (80%) treated for at least 3 concomitant diseases. The patients lived at home. Fifty-two percent of them lived alone. Sixty-seven percent of the patients needed additional at home nursing care. The usual treatment duration at the day-hospital is 28 days. The aim of the treatment is to improve and sustain a quality of life at home and empower the patient to be active in his social network. The most common diagnoses were cardiovascular diseases such as congestive heart failure (60%), falls (65%), arthrosis associated with chronic pain (50%) followed by other disorders, mostly polyneuropathy, diabetes, chronic obstructive pulmonary disease, malnutrition, urinary incontinence. The patients were offered group and individual interdisciplinary and medical treatments. The patients recruited from geriatric psychiatric day-hospital were mostly treated for mood disorders (70%), anxiety disorders (20%) and personality disorders (10%). The participants, received as usual outpatient care, including medical visits, psychotherapy and nursing care during their presence in the day-hospital. The usual treatment duration at the day -hospital is between 30–50 days. The main aim of the treatment is to maintain the patient in his natural surroundings and empower him to have an active role in society.

The participants received as usual outpatient care, including, medical appointments and nursing care. Participation in the study was voluntary and all participants were selected by convenience sampling when they came to the clinic for treatment. All patients were 65 years old and above.

Exclusion criteria were the followings: 1) severe cognitive impairment or mental retardation; 2) Mini Mental State score ≤ 24/30; 3) inability to communicate in French language; 4) Severe behavioural and mental health disturbances; 5) drug and alcohol acute intoxication or withdrawal.

The participants were administered the measures at one-time point in a face-to-face interview with a trained psychologist or psychiatrist.

All participants gave written informed consent. The study was approved by the Ethical committee of the Geneva University Hospital.

### Measures

Participants completed the following assessments:

 The French version of the ASSIST V3.0.[14]

 The French version of the Addiction severity index (ASI) [15]. ASI is an interview assessing history, frequency and consequences of alcohol and drug use. In the present study, the subsections related to lifetime and recent drug and alcohol use (3 months) were used.

 The French version of the AUDIT [16]. This auto-questionnaire is a reliable and valid tool for screening alcohol abuse and dependence.

 The French version of the Revised Fagerstrom Tolerance Questionnaire-Smoking

 (RTQ) [17] measures nicotine dependence.

 The French version of the Mini-International Neuropsychiatric Interview (MINIPlus)[18].

In the study at hand, the sections of the MINIPlus related to drug and alcohol abuse and dependence (lifetime and past 12 months) were administered.

This allows eliciting the presence or absence of dependence and/or abuse for: (i) alcohol (ii) the two other most frequently used drugs by the participants as assessed by ASSIST.

Furthermore, in order to exclude from the study patients with severe mental impairment, the participants also underwent a screening with the MINI MENTAL STATE questionnaire [19].

### Data analysis

Data was analyzed with SPSS (version 18.0, IBM, Chicago, USA) Proportions, mean values and standard deviations were used to describe the baseline characteristics for each group. We investigated the psychometric properties of ASSIST V3.0 by studying its criterion validity and construct validity. Criterion validity comes in two forms: concurrent validity where the new scale and the criterion are administered at approximately the same time and predictive validity where the new scale is meant to predict some later criterion [20]. We used the concurrent-type validity since the ASSIST was simultaneously administered with other existing validated instruments or administered within a short time of one another. Several domains or scores derived from the ASSIST together with scores from other questionnaires namely, the AUDIT, the MINI-Plus, the Fagerström and the ASI were used in the validation process. The ASSIST consists of eight questions, covering ten substances: tobacco, alcohol, cannabis, cocaine, amphetamine type stimulants (ATS), inhalants, sedatives, hallucinogens, opioids and “other drugs”. Test results are reported as significant for *p* < 0.05.

The correlations between the ASSIST domain scores and other similar instrument scores were assessed using Pearson’s correlations. For instance, the ASSIST tobacco scores were correlated with Fagerström scores and ASSIST alcohol scores were correlated with AUDIT.

ASSIST specific substance involvement (SSI) scores for each substance were also compared in the presence or the absence of MINI-Plus diagnosis of current or life-time abuse or dependence. A, two-tailed t-tests (or Mann–Whitney tests) were used where assumptions of variance homogeneity were violated. Finally, the total substance involvement score, excluding tobacco, was compared to the total number of MINI-Plus diagnosis which comprised the sum of current and life-time diagnosis of abuse and dependence for all drugs except tobacco. Internal consistency refers to the extent to which the items are interrelated. Cronbach’s coefficients were computed. This index varies between 0 and 1 and translates a greater degree of homogeneity of the items if its value is close to 1. Actually, its computation is based on the number of items on the survey and the ratio of the average inter-item covariance to the average item variance. It is generally accepted that the internal consistency of an instrument is satisfactory when the value of the coefficient is above 0.70 but not much higher than 0.90 [20]. The ASSIST was investigated for its ability to discriminate between three groups: non-problematic use, abuse and dependence. Clinically these three groups reflect the risk status of patients – that is, low, moderate or high risk. Risk status is proportional to the ASSIST score achieved. For a specific substance, people were classified in the low risk group if they scored 0 on the MINI-plus current abuse and dependence diagnosis (patient without substance abuse nor dependence). They were classified in the moderate risk group if they scored 1 for current abuse and 0 for current dependence. Finally the high risk group was composed of people with 0 for current abuse and 1 for current dependence diagnosis. Specific substance scores were compared using independent groups’ analysis of variance (ANOVA) with Games-Howell’s post-hoc test. Games-Howell does not assume that sample variances are equal or that sample sizes are equal. Hence it is a good alternative if this turns out to be the case. The same groupings were also used to perform receiver operating characteristic (ROC) analysis in order to obtain further information concerning the ability of the ASSIST to discriminate between groups.

## Results

### Sample characteristics

One hundred (100) patients, of whom 42 were from community medicine and 58 from general psychiatry, were interviewed for this study. Of this total, 28% were men. Age ranged from 65 to 93, with a mean of 77.8 ± 7.5 years. Except for age and nationality, no other statistical differences between groups were observed (Cf. Table 2). Patients with cognitive impairment (MINI Mental State ≤ 24/30) were not included in the present study.

**Table 2:** Socio-demographic and addiction data by place of recruitment

	**Total**	**Community medicine (n = 42)**	**General psychiatry (n = 58)**	*p*-value*
Mean age in years (± DS)	77.8 (7.5)	81.2 (6.3)	75.4 (7.4)	<0.0005
Gender, n (%)				0.09
- male	28	19	34.5	
- female	72	81	65.5	
Civil status, n(%)				1
- married	38	38.1	37.9	
- single/divorced/separated/widow	62	61.9	62.1	
Nationality, n (%)				0.002
- Swiss	75	88.1	60.3	
- other	25	11.9	39.7	
Higher school grade, n (%)				0.7
- elementary school	39	35.7	41.4	
- apprenticeship	21	19	22.4	
- secondary school and higher	40	45.2	36.2	
Mean AUDIT score (± DS)	2.9 (4.8)	3.3 (5.1)	2.7 (4.6)	0.5
Mean ASSIST score (± DS)				
- tobacco	2.3 (5.3)	2.4 (6)	2.2 (4.9)	0.8
- alcohol	3.7 (5.3)	4.3 (6.4)	3.3 (4.4)	0.4
- Cannabis	0. (0)	0	0	
- Cocaine	0. (0)	0	0	
- ATS	0. (0)	0	0	
- Inhalants	0. (0)	0	0	
- Sedatives	0.2 (1.1)	0.3 (1.6)	0.05 (0.4)	0.2
- Hallucinogens	0. (0)	0	0	
- opioids	0. (0)	0	0	

*Obtained by Student *t*-test or chi-square test/Fisher exact test respectively.

### Concurrent validity

1) Comparison with the Addiction Severity Index (ASI), Audit and FagerströmASSIST scores for alcohol had large positive correlations with the ASI and the AUDIT scores: (r = 0.73 and *p* < 0.0005; r = 0.8 and *p* < 0.0005 respectively). ASSIST tobacco scores also showed a large positive significant correlation with the Fagerström test (r = 0.8; *p* < 0.0005). No correlation could be computed for ASSIST and ASI opioids scores due to a lack of information.

2) Comparison with MINI-PlusParticipants recording current or life-time abuse or dependence diagnosis on the MINI-Plus had significantly higher ASSIST scores for alcohol compared with those for whom the same diagnosis was absent (Table 3). It is worth noting that out of the 19 participants with alcohol use disorder, 63.2% were females with an ASSIST mean score of 12.8 ±9.4 compared to 4.3 ± 5.9 for men.

**Table 3:** Comparison of ASSIST specific substance scores according to the presence or absence of MINI Plus current or life-time diagnoses of abuse or dependence

**Mean ASSIST scores by substance type mean (SD), n**			**T-value (*p*- value)**
	Diagnosis present:	Diagnosis absent:	
Tobacco	na	na	na
Alcohol	10 (8.5), 19	2.2 (2.6), 81	-3.9 (*p* = 0.001)
Cannabis	0 (na), 1	0 (0), 99	na
Cocaine	na, 0	0 (0), 100	na
ATS	na, 0	0 (0), 100	na
Inhalants	na, 0	0 (0), 100	na
Sedatives	na, 0	0.2 (1.1), 100	na
Hallucinogens	na, 0	na, 100	na
Opioids	0 (na), 1	0 (0), 99	na

na: sample size too small to allow comparison or information not available from questionnaire.

Besides, the total substance involvement score (without tobacco) correlated well with the total number of MINI-Plus diagnosis (r = 0.71 and *p* < 0.0005).

### Construct validity

#### *Internal consistency*

The ASSIST questionnaire was found to have a good internal consistency for the Global continuum substance risk score or the total substance involvement score (TSI) with a Cronbach’s α coefficient of 0.72 (95% CI ∈ [0.63, 0.79], *p* < 0.0005). Moreover, ASSIST scores for alcohol, tobacco and sedatives also showed moderate to good internal consistency (0.66, 0.74 and 0.89 respectively). The calculation of Chronbach’s α for the other substances was not possible due to insufficient data.

#### *Discriminative validity*

Table 4 shows specific substance scores grouped by diagnosis for non-problematic use (low risk), abuse (moderate risk) and dependence (high risk). Valid ANOVA results could be produced only for alcohol and not for the other substances due to insufficient data.

**Table 4:** Comparison of ASSIST domain scores for non-problematic use, abuse and dependence

**Domain**	**Use (low risk)**	**Abuse (moderate risk)**	**Dependence (high risk)**	**F-value, p-value**	**G-H**
	**Mean (SD), n**	**Mean (SD), n**	**Mean (SD), n**		
**3A**: SSI score for tobacco	na^a^	na	na	na	na
**3B**: SSI score for alcohol	2.9 (3.2), 89	na	22.7 (9.6), 4	-3.5, *p* < 0.0005^b^	na
**3C**: SSI score for cannabis	0 (0), 100	na, 0	na, 0	na	na
**3D**: SSI score for cocaine	0 (0), 100	na, 0	na, 0	na	na
**3E**: SSI score for ATS	0 (0), 100	na, 0	na, 0	na	na
**3F**: SSI score for Inhalants	0 (0), 100	na, 0	na, 0	na	na
**3G**: SSI score for sedatives	0 (0), 100	na, 0	na, 0	na	na
**3H**: SSI score for hallucinogens	0 (0), 100	na, 0	na, 0	na	na
**3I**: SSI score for opioids	0 (0), 100	na, 0	na, 0	na	na

^a^information not available from questionnaire or unable to perform post hoc test because there are fewer than 3 groups.

^b^comparison by Mann–Whitney test because only 2 non-empty groups and **too** small sample size.

Discrimination between use and abuse and between abuse and dependence could not be further investigated by ROC curves because there were no valid observations. As a result, estimation of sensitivity and specificity of the instrument could not be performed.

## Discussion

The results of this study indicate that the French version of the ASSIST is an acceptable and valid screening test for substance abuse and dependence in the short elderly sample. The findings are convergent with previous works on the validity of the ASSIST as a screening instrument for substance use disorders [7,13,14].

Concurrent validity of the French version of the ASSIST V3.0 was demonstrated by significant positive correlations between ASSIST scores and ASI, MINI-Plus, AUDIT and RTQ. The ASSIST has a good internal consistency, Cronbach’s α, ranging from 0.66 to 0.89.

The present, results show that ASSIST is a good and potentially useful instrument in general and psychiatric elderly health care settings.

This finding seems to be of great interest for clinical settings, considering the usually under screening of substance use disorders in elderly [2,5].

In comparison to previous screening-instruments, ASSIST has several advantages such as :

1. Screening for a wide range of substance use disorders. This is an advantage in comparison to others instruments such as the AUDIT [16] or the RTQ [17].

2. Short duration time of the questionnaire and a user-friendly aspect. This is an advantage in comparison to instruments like (ASI) [15].

3. Detection of substance use, abuse and dependence. This characteristic may be of particular interest with elderly helping to brake barriers, highlight the difficulties and possibly taboos related to the investigation of substance use disorders in elderly.

The present study has several limitations, such as the moderate sample size, a sample recruited only in University public outpatient facilities, the rarity of certain substance use in this sample, therefore, the estimates for sensitivity and specificity, for a number of substances could not be calculated. Furthermore, the study sample was not a general population sample. Future studies on large samples may be of great interest for the assessment of substance use disorders in primary care facilities and for further investigation of the ASSIST instrument.

The study presents however these findings for alcohol, the substance most widely used (19 participants have abuse or alcohol dependence). Despite the rarity of a number of substances misuse in at least the present sample, screening with ASSIST offers the advantage to check rapidly for a wide range of substances which is an important value added aspect in comparison to other screening instruments. These characteristics would be of great interests considering the lack of well validated drug misuse screening instrument for the aged [21] and also in consideration of the importance of alcohol and drug misuse in the elderly and particularly among the aged with comorbid psychiatric disorders [22].

Despite these limitations, our results suggest that the French version of the ASSIST could be used as part of a more general public health approach to the screening of substance use disorders in the elderly healthcare facilities. Further studies may assess impact of linking therapeutic interventions such as brief interventions to ASSIST screening in elderly people. As suggested by the WHO, this may help to reduce the burden of substance use disorders which are important risk factors of ill-health [7].

## Competing interests

The authors declare that they have no competing interests.

## Authors’ contributions

RK, YK and DZ designed the study. AC conducted the statistical analyses. RK, AC, YK and DZ wrote the first draft and compiled the co-authors' suggestions. All authors participated in the drafting of the manuscript and approved the final version.

## Funding source

Funds made available from the division of quality university hospitals Geneva.
